# Supplementary material for: The interactome of intact mitochondria by cross-linking mass spectrometry provides evidence for coexisting respiratory supercomplexes
Source: Mol Cell Proteomics. 2017 Dec 8;17(2):216–32. doi: 10.1074/mcp.RA117.000470 (PMC5795388; doi:10.1074/mcp.RA117.000470)
Supplement: Supplemental Data [file supp_RA117.000470_133922_0_supp_23978_fzffwf.zip › spectra_annotation/mito_spectra_annotation/340-1-4-1-10-1.pdf]

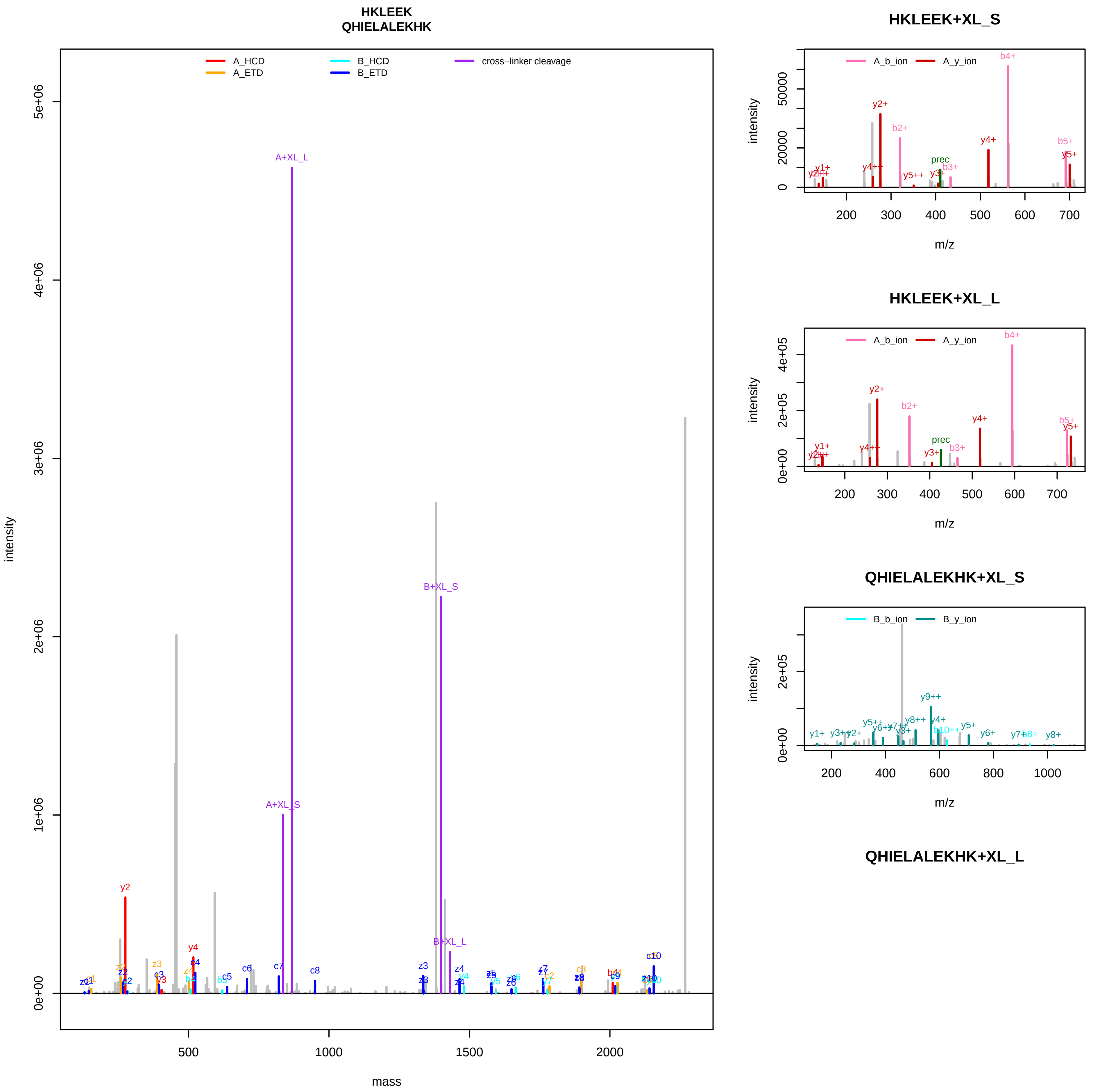
HKLEEK
QHIELALEKHK
A_HCD
A_ETD
B_HCD
B_ETD
cross−linker cleavage
A+XL_L
A+XL_S
B+XL_S
B+XL_L
intensity
mass
0e+00
1e+06
2e+06
3e+06
4e+06
5e+06
500
1000
1500
2000
HKLEEK+XL_S
A_b_ion
A_y_ion
b4+
y2+
b2+
y4+
b5+
y5+
prec
b3+
y3+
y1+
y4++
y5++
m/z
HKLEEK+XL_L
QHIELALEKHK+XL_S
B_b_ion
B_y_ion
y9++
QHIELALEKHK+XL_L
